# Multiteam Systems Handling Time-Sensitive Targets: Developing Situation Awareness in Distributed and Co-located Settings

**DOI:** 10.3389/fpsyg.2022.864749

**Published:** 2022-08-04

**Authors:** Thorvald Hærem, Sigmund Valaker, Eric Arne Lofquist, Bjørn Tallak Bakken

**Affiliations:** ^1^Department of Organisation, Inland School of Business and Social Sciences, Rena, Norway; ^2^Defence Systems, Norwegian Defence Research Establishment, Kjeller, Norway; ^3^Department of Leadership and Organizational Behaviour, BI Norwegian Business School, Oslo, Norway

**Keywords:** multiteam systems, communications condition, performance, situation awareness, time sensitive targeting, shared situation awareness

## Abstract

There is an increasing interest in how to organize operations carried out by multiteam systems (MTS). Large MTS typically operate with a dedicated integration team, responsible for coordinating the operation. We report a study of a military multiteam system that prosecute time-sensitive targets. We asked whether and how the integration team’s efficiency depends on its communication setting. Specifically, we studied how a co-located vs. a distributed communications setting influenced the shared situation awareness and whether the shared situation awareness again influenced the outcome of the decision processes. We found that performance fell when the integration team shifted from a co-located to a distributed setting. The fall in performance seemed to be mediated by a corresponding fall in situation awareness. Moreover, while the performance improved for each run in the co-located setting, we did not see such learning in the distributed setting. Qualitative observations revealed that misunderstandings lasted longer in a distributed configuration than in a co-located setting. We found that situation awareness at level 3 was the only level of situation awareness significant for predicting all dimensions of performance. Implications for theory, research, and practice are discussed.

## Introduction

Managing multiteam systems (MTS) is a current challenge for practitioners and organizational theorists. A fundamental assumption is that coordination through mutual adjustment within and among the teams in the MTS can be too costly (Davison et al., 2012). MTSs create integration teams to improve coordination and reduce the associated cost (de Vries et al., 2021). However, such integration teams are not always structured in the same way. This article focuses on large crisis management operations carried out by MTS, coordinated by an integration team where the team members are either co-located or geographically distributed.

Such MTS handle time sensitive targets, which is a special case of MTS tasks–concerning the “prosecution of enemy targets that require an immediate response because they pose, or will soon pose an imminent danger to friendly operations…” (NATO, 2008, p. 19). When a time-sensitive target is detected, decision-makers within a TST-cell have only a short time to gain a shared understanding of the threat, voice concerns regarding potential adverse outcomes, and coordinate the availability of assets to avoid the adverse outcome. Both theory and practical advice suggest that MTS tasks should be clearly defined, and information conveyance and processing standardized accordingly (Daft and Lengel, 1986; Wilson et al., 2007; Dennis et al., 2008; Firth et al., 2015). Other streams of research indicate that such standardizations have their limitations; Procedures for emergency responses provide clear guidelines for which actions to make and in which sequence. While it is often suggested that such TST-teams can efficiently operate geographically distributed using modern technologies to communicate and coordinate, experience shows that such distributed networks are associated with fatal misunderstandings (e.g., Wilson et al., 2007; BBC, 2016).

Empirical studies of crisis management demonstrate that the reality does not always fit the standard operating procedures (SOPs) and that crisis management teams deviate from their SOPs. This is often perceived as a paradox – since crisis management organizations take pride in their SOPs. Organizations deviate from standardized routines, even in simple environments (e.g., Pentland et al., 2010). The neatly organized sequential and pooled interdependencies suggested by military procedures–minimizing the need for communication by standardizing–do not describe the enacted interdependencies. When plans meet unexpected realities, individual responses result in enacted patterns of interdependencies that are both intricate and reciprocal (e.g., Ven et al., 1976; Hærem et al., 2015). Consequently, information needs to be integrated across several teams to manage the unexpected, by integration teams capable of mutual adjustment (Davison et al., 2012; Weick and Sutcliffe, 2015). de Vries et al. (2021, p. 6) found that co-locating operation centers helped increase understanding of other teams, encouraged discussion and questioning between teams, enabled socialization and sharing activities, and reduced silo-thinking.

Recent research has emphasized the importance of understanding integration in MTS as differentiation of goals, knowledge, and working practices increases (e.g., DeChurch and Zaccaro, 2010; Jiménez-Rodríguez, 2012; Larsen et al., 2014; Waring et al., 2020a). Others emphasize the need to study how the communications condition influence MTS coordination (e.g., Shuffler et al., 2015; Rico et al., 2018) and practices that improve the accuracy and timeliness of information sharing (Waring et al., 2020a). Modern communications technology has changed how multiteam operations can be coordinated and holds potential to facilitate distributed operations. However, the reliance on live video feeds and new text-based chat tools has also led to more confusion, misunderstanding (Cramton, 2001) and, less efficient communications processes–sometimes leading to both slower and more erroneous decision-making processes (Daft and Lengel, 1986; Wilson et al., 2007; Curtis et al., 2017). The conditions for achieving mutual adjustment may thus vary considerably between different MTS, and we explore a hitherto understudied aspect of integration teams in MTS: the role of communication condition.

Information differs as to the ease with which it can be shared, managed, and understood across different media (e.g., Daft and Lengel, 1986). For example, factual information can be more easily defined, structured, communicated, and understood in lean-media conditions than information related to deliberations of ambiguous situations. Although common structures, e.g., frame-of-reference training and shared standard operating procedures (SOP), can aid the development of shared understanding (Firth et al., 2015; Waring et al., 2020b), the effectiveness may differ between media conditions. What is missing in the extant literature on the link between communications contexts and performance in MTS is a deeper insight into the process that mediates the effect of communications contexts on performance and a deeper understanding of the role of various communications and information systems. Specifically, we investigate the influence of media conditions on a key organizational cognition construct, namely, shared situation awareness (SA) among component teams (Mohammed et al., 2021).

Situation awareness is basically about knowing what is going on around you (Endsley, 2000). More concisely, Jones and Endsley’s (2004) define situation awareness at three levels: The first step in achieving SA is to perceive the status, attributes, and dynamics of relevant elements in the environment (Level 1). SA, level 2, goes beyond the simple understanding of the present elements to include understanding the significance of the chosen elements in light of one’s goal. It includes how people combine, interpret, store, and retain information. The ability to project the future actions of the elements in the environment determines SA at level 3. SA level 3 requires knowledge of the environment and comprehension of the situation (both SA levels 1 and 2).

We argue that these levels correlate with increasing complexity and ambiguity, making communicating and sharing levels two and three SA more difficult. Since MTS coordination requires the integration of specialized teams operating at different geographical locations, we ask; whether and how the communications condition influences the establishment of SA in a MTS and thereby, potentially, affects performance?

This study was a part of an organizational development project to improve the organization’s management of MTS in TST operations. The organization tested two ways of organizing such operations; one with a co-located TST-cell and one with a distributed TST-cell.

### The Context Studied; the Time Sensitive Targeting-Cell as a Multiteam Systems Integration Team

Time sensitive targeting-cells are what scholars on multiteam systems name “integration teams,” teams that are formally “tasked with coordination and system integration” (Davison et al., 2012, p. 809; de Vries et al., 2021). The context of decision-making in a TST-cell has similarities to decision-making in many other High-Reliability Organizations (HROs), such as aircraft carrier flight operations at sea, nuclear power plants, flight controllers, and oil drilling and production platforms (Rochlin et al., 1987). The TST-cell integrates the input of various teams needed to conduct such operations: planning, intelligence, supervision of multiple forces, etc. These are highly specialized teams with functionally different roles that are dependent on each other to complete their mission. Only through efficient integration is it possible to match and adjust information and plans with actions effectively. The high-reliability settings share characteristics, such as, the use of clear procedures guiding decision-making, and deviations from procedures may have catastrophic consequences. However, TST-cell decision-making differs from these HROs in crucial aspects. While HROs typically have core operations that work reliably to avoid crises, reliably handling crises is the core operation, not the exception, in a TST-cell.

A TST-cell represents a large and diverse multiteam system representing different teams within different domains of expertise and capabilities. The TST-cell coordinates and integrates the efforts of these specialized teams. Due to the high complexity of the operations, the high stakes, and the time-sensitivity, the need for accurate and timely information exchange between the specialized teams is critical. Such MTSs (McCrhystal et al., 2015) are defined as networks of interdependent teams that coordinate to achieve shared SA (Zaccaro et al., 2020). The TST-cell sets the ultimate coordinative agenda. It shares goals, subgoals, and corresponding sub-processes within the differentiated teams (Firth et al., 2015). The TST-cell could be likened to a hub in the network of teams, channeling information among sub-teams (e.g., intelligence about an enemy and the status of own forces) so the MTS can act in concert.

The multiteam system we studied had implemented a version of the SOP for TST developed by NATO (2008). One purpose of the SOP was to facilitate that the whole MTS knew the exact goals the TST-cell sought to accomplish. The main phases of the SOP for time-sensitive targeting were (1) Target Data Collection, (2) Mission preparation and SA production, (3) Find, Fix, and Track Target, (4) Engagement of the target, and (5) Assessment of the engagement. As explained below in the Section “Materials and Methods,” we focused primarily on the first three subgoals/sub-processes. The SOP for the TST was essential to facilitate the coordination among teams by explicitly making the members aware of the interdependencies inherent in the phases of the TST. The interdependencies in a MTS will vary in importance over the phases of the decision-making process (Firth et al., 2015).

The TST-cell can be analyzed as a dedicated integration team (Davison et al., 2012, p. 809; de Vries et al., 2021) for a large multiteam system operating resources from the Air Force, Navy, Army, and Intelligence. The team is a team of teams designed to quickly and efficiently respond to and handle highly prioritized threats. The TST-cell we studied was composed of the following roles, each representing a component team dedicated to performing differentiated and specialized services: (1) The TST-cell Chief (CC) led the integration team; (2) the second in command (XO); (3) a senior intelligence duty officer that was responsible for integrating information from the intelligence surveillance and reconnaissance team (SIDO ISR); (4) a senior Operations Duty Officer (SODO tgt) that was responsible for targeteering and weaponeering (i.e., assigning weapons to specific targets); (5) a TST-CC Legal Adviser (Legad) providing the available judicial resources; (6) a battlefield coordination element/Special Operations liaison element (BCE/SOLE) that was responsible for input from the Land and Special Operations forces; (7) a role that was responsible for input from the maritime forces (MCE); and (8) a Communications Officer (COMMS).

The information required to solve each phase in NATOs SOP for time-sensitive targeting creates interdependencies among all the participating teams. The representatives were liaisons from their component team and were in close contact with the component team they represented. The CC and his subordinate (XO) were part of the collocated Air Operations Center. The SIDO was connected to the intelligence team to receive live intelligence. The SODO tgt represented the planning team, while the maritime liaison (MCE), and the Legad represented the legal team in the Joint Operations Center. BCE/SOLE represented the ground forces and special operations teams, and MCE represented the maritime operations team in the Joint Operations Center.

The different teams had special responsibilities in different phases of the mission. For example, the intelligence team would be vital in the target data collection phase. In phase 2, this team could collaborate with the targeteer team to plan the mission and prepare the rest of the MTS for the Find, Fix, and Track Target phase. Specific assets such as surveillance aircraft, fighter aircraft, and naval vessels with sensors could be needed to accomplish phase 3. The legal team had to continuously react to changing premises to ensure that the TST-operation stayed within the legal boundaries.

In a planning mode, most dependencies can be sorted sequentially (Thompson, 1967). As unexpected events and actions occur within the MTS, the many sequentially ordered dependencies will shift to reciprocal and, thus, require mutual adjustments (Thompson, 1967). This increases the coordination demands on the MTS, and the role of the integration team becomes even more critical. The multiteam system will be distributed and operate at different places. The integration team, however, may choose between a distributed and co-located organization. This choice is a core issue of much theory on team coordination (Okhuysen and Bechky, 2009) and also in this study.

Specific technologies have been developed to coordinate distributed MTS doing TST (Orhan and Yakup, 2013). Being distributed allows each participant to focus on their task and, less disturbed, coordinate with their subordinates. The multiteam TST-cell we studied had implemented a range of communications technologies specially designed to support TST decision-making processes and utilized the advantages of a distributed team of teams. These tools provided several benefits over face-to-face communications. The chat tool provided a continuous memory of what had been said by whom to whom. The tools also kept a clear log on all tasking orders and the disposition of resources. Based on such technological enhancements, there were good reasons to ask whether distributed TST-cells may produce better SA, self-synchronization, and performance than co-located TST-cells.

## Theory

We first discuss how communication media influences the performance of an integration team in a multiteam system. Then we discuss how media affects SA, and lastly, we discuss the influence of SA on the performance of the integration team.

### Media Condition and Its Influence on Integration Team Performance and Situation Awareness

Uitdewilligen and Waller (2012) argue that in an MTS, “richer communications channels” are required to facilitate the integration and interpretation of information. Media richness theory (Daft and Lengel, 1986) has been used to analyze the richness of a communications setting according to its ability to change situation understanding within a given time. In a multiteam system, such as MTS prosecuting time sensitive targets, the dynamic updating of shared SA is critical but challenging since the team often operates at different locations. Media richness theory proposes that effective communications require a match between the task and the communications setting. Lean media should be utilized for tasks low in complexity (distributed text-based communications condition) and rich media (co-located communications condition) for tasks high in complexity. The theory also proposes that there would be an overkill to use rich media to solve tasks low in complexity and equally ineffective to use lean media to solve tasks high in complexity.

Meta-analyzing the research on team virtuality, Ortiz de Guinea et al. (2012) found that co-located settings correlated with better information sharing, leading to better team performance outcomes. Adding nuance, Mesmer-Magnus et al. (2011) found that distributed teams were better than co-located teams at sharing unique information but worse at open information sharing. Although prior research has produced mixed findings, the media richness tradition and the media synchronicity tradition (e.g., Dennis and Kinney, 1998) suggest that a co-located setting positively influences performance in a TST setting with dynamic ambiguity and complexity.

A good decision process may not always lead to a great outcome, and a great outcome may not always be the result of a great process; coincidences, luck, and noise often play a role. Therefore, we evaluated both the process and the outcomes to evaluate performance. To capture process performance, we assessed how each of the central points from the TST standard operation procedure that NATO developed performed. See Cetinkaya and Yildrim (2013) for a detailed description of the SOP. We focused on the first three general phases of the procedure (1) Target Data Collection, (2) Mission preparation and SA production, (3) Find Fix Track Target, and (4) engagement of the target. Regarding outcome, TST-cell performance is defined as the accuracy of the decision (performance accuracy) and how quickly the decision was made (performance speed). Defining performance, both in terms of the process and outcome (accuracy and speed), is also in line with the team literature (Beersma et al., 2003; Burke et al., 2006).

We suggest that both process and outcome will benefit from a co-located setting in the complex task carried out by a TST-cell. As indicated above, the information sharing needed to coordinate resources adequately often requires transmitting detailed information in a precise time interval. The timeliness of information may be critical to achieving accurate SA to coordinate a set of complex actions. The possibility of being co-located, exchanging verbal and non-verbal cues, and adjusting and negotiating meanings should help such activities (Cramton, 2001). On this basis, we will explore whether performance, both process and outcome (speed and accuracy), will be higher in a co-located than in a distributed setting.

### Media Condition and Shared Situation Awareness

A key element of integrating information and a premise for effective MTS coordination is achieving a shared SA (Luciano et al., 2018). Organizations often implement a Joint Decision Model or SOP to integrate and coordinate information for adaptive cognitive processing in the multiteam system (Waring et al., 2020b). The organization we worked with adapted the NATO SOP for the TST-process to coordinate and integrate SA. Common knowledge about the procedure and the criteria for moving from one step to the next should ensure that all members know what to achieve. One of the cell members argued that;

*”When such common knowledge is missing, the unknowing member is unable to contribute with precise information at the right moment and thereby contribute to the speed of the process. Team members lacking an accurate SA will both slow down the process and make the decision process less reliable.”*

Media synchronicity theory suggests that the ability of media to support coordinated actions and allow for turn-taking among team members is essential to form shared understanding (Dennis et al., 2008). Furthermore, the ability of rich media to enable accurate information sharing will be essential in developing a shared understanding through the added capability of rich media to aid in the contextualization of information (Te’eni, 2001). On these grounds, lean media would be ineffective when the uncertainty is high and might lead to prolonged decision-making processes and even incorrect decisions.

Early experience with the TST procedure called attention to the need for rapid development of shared understanding within the TST-cell. The required sharing of information from the liaison-specialists from the different component teams pointed to the central role of communications. Interpersonal communication seems central when integration of diverse areas of expertise is required and there is a requirement to update and make sense of the environment (Maitlis and Christianson, 2014). Marlow et al.’s (2018) meta-study of the team-communications–performance relation finds that information elaboration often is particularly productive in avoiding misunderstandings. Identifying the need for elaboration would be easier in a face-to-face setting because it facilitates signaling and detecting mental states such as uncertainty and confusion. The combined insights from our initial study and the research literature guided us to explore the effect of the communications condition more rigorously.

Endsley et al. (2003) defined shared SA as the degree to which team members have the same SA. In line with this definition and the three levels of individual SA, we define sharedness as the degree to which the team members share SA.

One may suspect that SA will vary across the roles in the team, and one may wonder which roles must share the common awareness–and which roles may have a less integrated SA to perform well as an integration team. How sharedness varies in a multiteam system is an under-researched topic (Endsley et al., 2003). Our operationalization of sharedness allows us to investigate how SA varies across roles and how individual roles’ SA co-vary with team performance.

Based on the predictions in the media synchronicity theory we would expect that the shared SA on level 3 will benefit the most from a co-located setting as the co-located setting better facilitates the processes for integrating information (Cramton, 2001; Dennis et al., 2008; Valaker et al., 2018). Conversely, the theory suggests that the shared SA, particularly level 3, will be challenging to develop in a distributed setting because it will be difficult for the integration team to converge on the more abstract thinking required for predicting future states of the operations (Valaker et al., 2018). A distributed setting will potentially delay and distort the integration of information required to form SA at levels 2 and 3 (Cramton, 2001; Valaker et al., 2018). On the other hand, we suggest that SA on level 1 will benefit from a distributed condition. The typically lean text media used in this context will be better at transmitting large amounts of information, such as a vast amount of identified objects (Valaker et al., 2018).

Furthermore, shared SA level 3 is particularly relevant to TST processes and performance. To the TST-cell, a sign of efficiency is to reduce the time to engage moving terrorist targets since it would enable “on-time” positioning of resources to intercept threats. Without the shared ability to predict the future state of the operation, and the terrorists’ actions, it would be impossible to position the required resources at the right place at the right time. Therefore, there are good reasons to expect that high SA at level 3 distinguishes successful runs from less successful runs.

### Learning to Acquire Shared Situation Awareness in the Co-located vs. the Distributed Setting

We combine Waring et al. (2018) with earlier theorizing of information processing in organizations (e.g., Daft and Lengel, 1986) to explore whether the MTS’s communications setting influences the ability to develop shared SA at the three levels. Information elements required to build SA1 are easier to identify and transfer than the elements necessary to create SA2 and SA3. SA2 can depend on SA1, and SA3 depends on first having developed SA1 and SA2. Going from SA1 to SA2 requires integrating SA1 type information, over time, to understand and observe, for example, whether two enemy objects are part of the same formation (e.g., enemy naval vessels being part or not of the same maritime action group). And based on SA2, different courses of action, e.g., routes that the enemy could take, may need to be simulated, and the likelihood of each course of action examined. Taken together, the characteristics of SA1, SA2, and SA3 suggest that they do not develop at the same speed. In addition, and as indicated by Cramton (2001), there may be significant delays in developing mutual understanding in distributed settings. Moreover, SA2 and SA3 reflect information requiring more complex integration and interpretation processes than SA1. While the factual information acquired for SA1 could lend itself to the clearly defined information gathering and processing according to subgoals in an MTS (Firth et al., 2015), the integration of information needed to creatively develop SA2 and SA3 lend itself more to the sensemaking process suggested by Weick et al. (2005). We argue that such sensemaking processes are better accomplished in a face-to-face setting. We, therefore, explored whether the generation of SA2 and SA3 is better in the co-located setting than in the distributed setting. We also investigated whether the creation of SA1 was better in the distributed setting than in the co-located setting.

## Materials and Methods

### Participants

The participants in the integration team were eight male officers from a joint-level headquarters in a NATO country. All had operational experience from prior TST exercises, and their military ranks ranged from lieutenant (O3) to lieutenant colonel (O5). In total, the team engaged in five tasks. The key variables, except performance, were measured three times in the four first tasks and four times in the fifth task. The repeated measures yielded a total number of observations of 128. As there can be a learning effect when the same team solves similar tasks over time, we ran the co-located and distributed settings every second run. We decided to let the teams start co-located so that the learning effect would favor the distributed setting most and the co-located setting least (Alge et al., 2003).

### Research Design

We designed an action research project with the NOBLE (Norwegian Battle Lab and Experimentation) organization, the NATO joint command, and three NATO military organizations; (land-, sea-, and air force). This article reports on the last part of the action research project. In the first phase, not reported here, we designed a TST-cell set up to coordinate the prosecution of time sensitive targets and identified human factors we believed were important to the outcome of the operations. In the last phase, we explored how these human factors played out when the TST- integration team for the MTS operated under two different conditions, one co-located and one distributed. The scenarios reflected realistic operational challenges and were run over 3 days.

As described below, we systematically observed and evaluated the process and outcome of the TST-cell operations. Four observers assessed the performance of the process. Three of the observers were high-ranking military specialists within relevant areas of specialization. The fourth observer was a civilian working with military decision-making processes for 3 years. After each run, the observers wrote a report of their qualitative assessment of the run. After each run, qualitative interviews were conducted to follow up on the observations made during the runs and helped clarify the observations’ validity. We collected questionnaire data for SA during each run and logged performance on predefined criteria.

### Procedure

The experiments ran over 3 days and included one TST-run on day one, two on day two, and two on day three. The runs were executed as one ongoing operation, with three to four windows of opportunity to take out the terrorist(s) in each run. In total, there were 16 observations of TST-cell performance across all the windows of opportunities across all runs. As the objective of the experiment was to compare the two different ways of organizing (co-located and distributed setup), the runs had to be similar. Therefore, we tried to create the runs as similar in character and complexity. However, it was important that the scenarios were not identical, possibly giving one form of communications setting an advantage due to learning curve effects. The runs were rotated between distributed and co-located settings to minimize the sequence effect. The complexity of the runs also increased each succeeding day. The experiments were controlled by a White Cell consisting of four observers. All inputs to the TST organizations were pre-scripted, and it was possible to pause the exercise at any time.

### Scenario Description

The main effort and mission of the military organization simulated during the experiment was the protection of a fictive nation’s Parliament inauguration ceremony conducted on the third day of the investigation. The main threat to the mission came from a fictitious terrorist organization.

### Data Collection, Operationalization, and Measures

#### Communications Condition: Co-located and Distributed Time Sensitive Targeting-Cell

The experiments were run in the same facilities as those used in an actual situation. In the co-located condition, all of the critical members of the TST-cell were present, face-to-face, in the operations room, and they could phone members of their respective teams, either through a shared phone or through the comms role, which had access to a phone. The phone conversations were one-to-one and not heard by the whole room. The cell members stood around a Common Operational Decision Support (CODS) table showing a common operational picture of their air- and seaborne assets. The CODS table was a high-definition screen that displayed the air- and seaborne assets and their positions in a scalable map. The video stream from the UAV-sensors was projected on a screen for all the members to watch. The TST decision-making procedures (TST management tool) were also projected on a screen and the TST status board (whiteboard).

In the distributed cell, the members were in different locations and communicated through the NATO planning tool FAST and the NATO instant messaging system JCHAT. Each role had a phone that they could use to phone each other and their respective teams. They could access the common operational picture on their computers, accessing the same information as in the collocated setting regarding air and seaborne assets and their location displayed on a scalable map. The SIDO, intelligence manager, had access to the live UAV-feed.

The distributed cells were coded as 0, and the co-located as 1.

#### Performance; Process and Outcome

The performance process was operationalized according to NATO’s TST management tool procedures, reflecting how well the TST-cell performed at the different stages of the TST-process (Process performance): (1) Target Data Collection; (2) Mission preparation and SA production; and (3) Accomplishment of the find-fix-track-target steps in the TST process. *Find* means detecting and characterizing targets for further prosecution. F*ix* refers to determining the location and identification of the potential target. *Track* means observing and monitoring a target’s activity and movement. Target consists of determining which options are available for striking a target, maintain track, check if the target can be engaged, and decide whether to engage the target. The criteria for achieving each of these phases were specified as part of the scenario design.

The average of the observers’ scores was recorded as the TST-cell’s score on each of the four phases. The inter-coder reliability for process performance was assessed to be 0.75, indicating that the coders had a relatively high level of agreement about the TST-cell’s scores. The same observers collected data on outcome performance and speed. The inter-coder reliability for performance outcome and the speed of the operation was 1. All performance outcomes were scored from 0 (minimum) to 100 (maximum). For speed, we reversed the scoring such that lower speed was scored higher.

Performance, as an outcome, was operationalized by the degree to which the three to four windows of opportunity were utilized (Performance accuracy): A more accurate integration team took advantage of an earlier window of opportunity. We used clock time to measure Performance as the speed with which the tasks were solved (Performance speed).

#### Situation Awareness

Situation awareness (SA) was measured by a twelve-item questionnaire administered to the TST-cell members at the start of the last phase in the TST decision process of the engagement phase. We used the SAGAT (Situation Awareness Global Assessment Technique) to assess the awareness of the current situation by asking the integration team members questions about key aspects of the situation that could be objectively scored as correct or incorrect (Endsley, 1995, 1988). Level 1 was measured by four items, and levels 2 and 3 were measured by three items each. Building on SAGAT, we adapted the SA-questionnaires to the scenarios and the windows of opportunity. Level 1 questions asked about specific factual information such as the location and type of enemy resources and own resources, for example, *“What kind of effectors do we possess?”* For level 2, we asked factual questions about the relations between elements in the situation, like; *“What kind of military actions are possible for us to pursue in the present situation?”* Level 3 items gauged respondents’ understanding of the likely course of action of hostile objects. For example, *“What are the most likely terrorist preparations expected at “location 1”?”* It was possible to achieve a maximum of 100 points.

**FIGURE 1:**
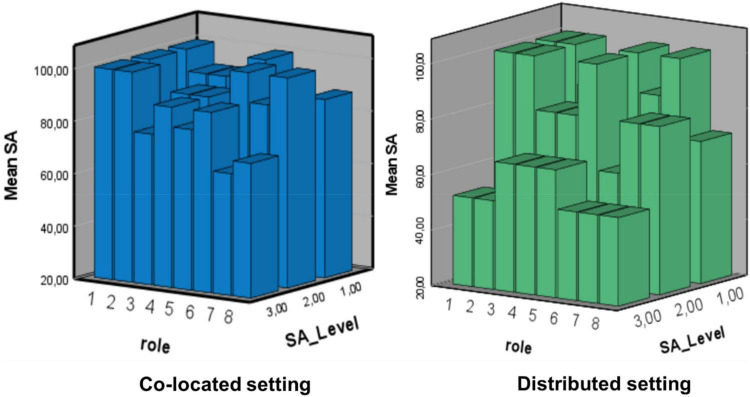
The sharedness of situation awareness (SA) across SA levels and across team roles. The x-axis represents the roles in the integration team from 1 to 8 as presented on page 4: (1) The TST-cell Chief (CC) led the integration team; (2) the second in command (XO); (3) a senior intelligence duty officer that was responsible for integrate information from the intelligence surveillance and reconnaissance team (SIDO ISR); (4) a senior Operations Duty Officer (SODO tgt) that was responsible for targeteering and weaponeering (i.e., assigning weapons to specific targets); (5) a TST- CC Legal Adviser (Legad) providing the available judicial resources; (6) a battlefield coordination element/Special Operations liaison element (BCE/SOLE) that was responsible for input from the Land and Special Operations forces; (7) a role that was responsible for input from the maritime forces (MCE); and (8) a Communications Officer (COMMS).

We calculated the individuals’ SA as an average of the items for each level. The team’s shared SA was computed as the average of the integration team members’ individual SA. This operationalization of shared SA does not capture the variation across the roles in the team. However, by plotting all team members’ individual SA we get a picture of the variation in the team across the two communication settings (see Figure 1).

## Findings

This section presents our analysis of the qualitative and quantitative data for the relations investigated. Table 1 below reports the descriptive statistics and the correlations among the main variables observed. The observations are of the eight military officers over six runs, producing 48 observations of SA. There were about three windows of opportunity (to capture the terrorists) for each scenario, so the number of observations of windows of opportunity was 144. The Communications condition (distributed = 0 and co-located = 1) correlates positively with process performance, performance speed, performance accuracy, and SA at level 3. The three types of performance correlate positively with each other. Level 3 SA correlates positively with the three types of performance, while SA at level 2 only correlates positively with performance accuracy. We also note that SA at level 1 correlates positively with SA level 2 but not with SA level 3 and performance.

**TABLE 1:** Descriptive statistics and correlations.

Variables	Mean	SD	1.	2.	3.	4.	5.	6.
(1) Communication condition^1^	1.56	0.50						
(2) Process Perf.	57.38	12.18	0.81*					
(3) Speed Perf.	–83.3	21.39	0.85**	0.62**				
(4) Accuracy Perf.	39.07	19.78	0.63**	0.31**	0.62**			
(5) SA 1	90.54	12.56	–0.04	−0.22*	–0.09	–0.11		
(6) SA 2	88.08	18.30	0.13	0.02	–0.10	0.25**	0.35**	
(7) SA 3	72.37	23.43	0.58**	0.41**	0.68**	0.70**	–0.04	0.03

*^1^Communications condition: Co-located = 1, distributed = 0.*

*p < 0.05, **p < 0.001.

*N = 48.*

For all performance dimensions, performance increased when the TST-cell was co-located. Most of the cell members said that in the co-located setting, information was processed faster, and the progress was easier to follow. In the co-located condition, the TST-CC used the process management tool (the SOP-check list) to guide the shifts from phase-to-phase to a greater extent than in the distributed cell. As theorized by D’Adderio (2011), the SOP-check list was an artifact that held the routine together and triggered shared interpretations of the process and situation. The shared visualization helped the cell members in the co-located cell to see where the TST-cell was in the decision-making process. The TST-CC’s use of the management tool to oversee and orchestrate the team seemed to facilitate a natural transmission from a sequential to a parallel processing of information. The cell members used the tool to recognize where they were in the different phases. The process management tool was used less when the TST-cell was distributed. Correspondingly, the speed and accuracy of the process fell as the setting shifted from co-located to the distributed setting.

The quantitative data supported these qualitative judgments. The quantitative results indicate that for performance accuracy, the co-located cell achieved, on average, about 50% higher performance scores than the distributed cell (an average score of 50 for the co-located runs and an average score of 25 for the distributed runs).

If the cell managed to be accurate enough to utilize the first window of opportunity to make a decision, a 100% score could be achieved. There were four windows of opportunity (to take out the terrorists) in each run, and the score degenerated linearly as windows were missed. If all windows were missed, the cell scored 0. No runs in the two conditions managed to utilize the first window of opportunity. In all runs, the distributed cell missed all but the last window of opportunity to get the terrorists.

The co-located cell condition was, on average, about 1.75 times faster than the distributed cell. The speed of the TST process improved over the runs, and the speed improvement was greater for the distributed cell than for the co-located cell, although the ability to take out the terrorists did not increase in the distributed cell, while it did improve in the co-located cell. To understand the mechanisms driving the differences in performance in the two conditions we first look at the generation of SA.

### Consequences of Communications Condition on Situation Awareness

First, it is interesting to note that, on average, the SA on levels 1 and 2 was not very different across the two ways of organizing the TST-cells. In fact, SA at levels 1 and 2 was about the same in the distributed cell as in the co-located cell. But, SA at level 3, the ability to predict the development of the situation, was considerably higher in the co-located cell than in the distributed cell. To understand the concept of sharedness of SA in more detail, we plotted the average SA measures, reflecting how the sharedness of SA varies across the roles and the three levels of SA in the co-located and distributed setting. The two cubes in Figure 1, below illustrate this.

There is a striking similarity and difference in the profiles of the sharedness of SA in these graphs. First, the similarity: Figure 1 illustrates well what we also inferred from Table 1: a considerable correspondence between SA levels 1 and 2 across the two settings. The scores are 100% for several roles in both settings. Role 1 reflects the Cell-Chief’s scores; he scores 100% across the three levels in the co-located setting and 100% on levels 1 and 2 in the distributed setting. Second, the striking difference is that the level 3 SA falls considerably for the whole team in the distributed setting. Scores at 25% indicate no better than a random response to the SA questions. We see that all the roles in the distributed setting have a substantial reduction in SA on level 3.

The high scores on SA1 and 2 in both settings, and the fall in SA3, indicate that getting facts is equally difficult or easy in the distributed setting as in the co-located setting, but integrating the information to a more complex shared SA was more difficult in the distributed setting. SA seemed to also interact with the degree of self-synchronization. The ability to give the correct information at the right time seemed important for SA level 3 and the ability to act based on that awareness.

Situation awareness level 3 is the ability to predict the development of the situation. Without this ability, the performance falls. The numbers in Table 1 underscore this relation–there is a strong positive correlation between SA3 and all performance measures. We asked the cell-members to elaborate on this observation. They pointed to the common operational picture in the co-located setting, which everybody could see, share and use as a basis for the discussions as a tool to avoid misunderstandings. The TST legal advisor said this about the co-located setting: “*It was possible to reach everybody simultaneously and gain immediate feedback from the cell members*.” The COMMS, commenting on the co-located TST-cell, said: “*I could see how the people reacted when they received information*.” In the interview after the last run, the Cell-Chief provided the following summary of the advantages and disadvantages of the co-located and distributed settings related to the development of SA presented in Table 2.

**TABLE 2:** Summary interview with Cell-Chief.

	Co-located	Distributed
**Situation awareness**		
Documentation of decision process	Not so good. Cannot go back and check information of what has been done. However, the information in the co-located cell is given in more vivid forms and one recalls information easier.	Very good, facilitates memory. Can go back and check information. Tracks history. However, when something (wrong) is posted, it is difficult to clarify it later (referring to a misunderstanding about the location of the terrorists), at least if it is not clarified right away.
Visualization	Common operational picture is better in Co-located. One can see the physical picture. The tasks are clearer and one can see what other members are concerned with and what they discuss.	The facilitation of the common operational picture is not good, and one does not see what other members are concerned with. There is a greater need to double-check information.
Communication	When you are co-located you can go in and ask just the right question. As I hear what they are saying, I can immediately take action and correct the way things are going. The co-location enhanced the possibility for immediate human feedback. A cell member’s body language can tell something about a person’s SA.	It is difficult to create a common SA in a distributed cell. I tried to compensate for that by writing on the chat how I see the situation just now. You do not really know what each cell-member is doing. I use the phone to clarify, correct or specify the assignment. In the chat you can see if the discussion is going in a wrong way. I tend to take a phone instead of writing many lines of elaborate explanations. I can also just delete a task if I see that things are going the wrong way.

As the Cell-Chief noted, we also observed that misunderstandings and errors were more persistent when they occurred in the distributed cell than in the co-located cell. In several incidents, erroneous information was entered into the log-system. This erroneous information was often not corrected immediately, and the corresponding misunderstanding often lasted throughout the run–inhibiting adequate responses from other cell members. This caused parts of the multiteam system to allocate the wrong resources to the wrong locations at the wrong time. Other media were available, such as telephones, but these were not used in these instances. One example is the following: In run two, day two, “Joint Operations Command–land” reports observations of the terrorist at the Ferry Quay, and that suspicious people were observed at the cottage 2 km east of Reitan Ferry Quay. Somehow, this information was transformed into “*Observed at least three suspect people at Naurstad sea/camp gathering outside the cabin*.” This information was erroneous, and much of the decision-making process became devoted to finding more information about three possible terrorists at the Naurstad sea/camp. The misunderstanding lasted the entire operation. Similar examples of misunderstandings were found in the transcripts from the chat and log-system.

In the co-located setting, similar misunderstandings occurred, but they were clarified much earlier. For example, when a similar misunderstanding occurred in run 1–day 2, the cell members stood around the CODS-table, pointing at locations at both sides of the bay, discussing different understandings, thereby clarifying the misunderstandings immediately. Another example from run 1–day 3 illustrates how a complex situation was handled using rich face-to-face communications in the co-located cell. Early in the run, during the mission briefing, the CC started to plan for the maritime units based on misunderstandings about the location and availability of the naval units. The advisor to the TST-cell on naval forces looked at the common operational picture and was uncertain about the correctness of the premises of the plan. The CC immediately sensed this uncertainty and initiated a discussion about the facts. The misunderstanding was quickly sorted out, and the planning for, and use of, the maritime units were corrected. These are examples of how factual errors in the SA on levels 1 and 2 lead to erroneous awareness on level 3 in the distributed setting.

All cell members agreed that the process went faster in the co-located TST-cell due to the better visualization and control of available sensors and effectors (fighter jets). Discussions were faster because writing in a chat takes more time than talking face-to-face. There were situations when some cell members felt that there was too much noise in the co-located setting, and lack of discipline occurred in some short periods. A cell member pointed out that some group pressure might have occurred, causing some cell members to give the green light without being 100% sure.

**FIGURE 2:**
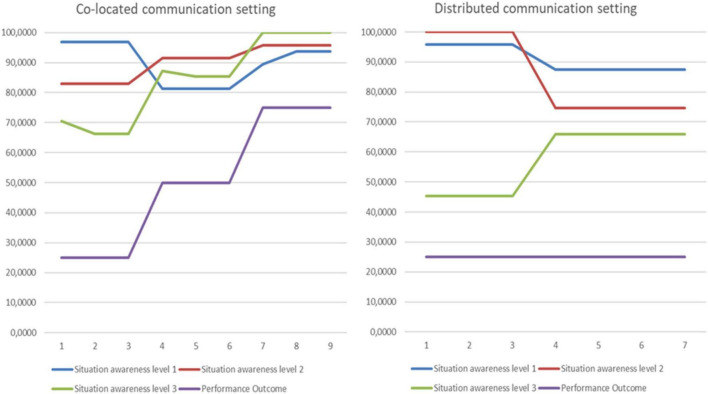
Characteristics of the runs in co-located and distributed settings.

While Figure 1 provides the average SA across all the runs, it does not consider the time dimension or the learning process. To see the time dimension, we plotted the development of SA across all runs and windows of opportunity (x-axis) in the figure below. We also included the performance indicator in this figure (Y-axis from 0 to 100%). In the distributed setting, the performance was constant across the runs. SA on level three improved to the level the co-located had on the worst run, while SA levels two and three did not improve. Although SA3 increased in both settings, it plateaued at a lower level in the distributed than in the co-located setting. SA2 decreased in the distributed setting, while in the co-located setting, SA2 increased. See Figure 2 below.

These charts (Figure 2) illustrate the difference in learning between the settings. Since it is the same team performing all runs in both settings, we cannot blame differences in skills and experience. The difference is likely due to the two settings’ technical setup and physical communication conditions. The communications difficulties pointed to by the cell-members provide some cues for our discussion.

## Discussion

The main question in this study was whether and how the communications condition influences the establishment of SA in a MTS and, potentially, thereby affects performance? All the measures of performance fell when we shifted from a co-located to a distributed cell. The process performance was remarkably reduced. We found that a distributed cell did not influence SA compared to a co-located cell on SA levels 1 and 2 but significantly reduced shared SA on level 3. SA levels 2 and 3 increased in the collocated but decreased in the distributed setting. Qualitative observations indicated that misunderstandings lasted longer in a distributed than in a co-located setting. We tentatively attribute this to the selective use of available communications media: not shifting from text to voice communication inhibited rapid feedback and clarification. Moreover, in the distributed setting, the participants did not see whether or what the others looked at in the common operational picture. In the co-located setting, the mutual acknowledgment of what one saw in the common operational picture was much more immediate. It could have reduced the risk of misunderstandings or facilitated the correction of misunderstandings. Finally, we found that SA at level 3 was the only level significant for predicting performance on all dimensions of performance.

We found that the SA in the distributed condition did not fall at levels 1 and 2, but for level three–it fell dramatically across all runs, for all the roles, indicating that integrating information was more difficult in the distributed setting. Looking back, we notice that the key elements we asked about at levels one and two were often all correct–but the team generated their own “facts” endogenously by a process of misunderstanding. The misunderstandings undermined their shared level three SA and, thereby, their performance.

To be clear, the misunderstanding and the errors did not come from external complexity in the scenarios but came from the faulty processing of initially correct information. But why were the errors corrected only in the co-located setting? The probable reasons for this difference can be traced to the inherent properties of the two communications conditions. When information was entered into a written media format, most of the uncertainty attached to the information was censored by the communications condition. In the distributed setting, the information receiver took the written information at a higher “face validity” than when the information was given face-to-face. Added to this, the preference for text messaging and the chat system resulted in less active use of the voice tools available.

The increased authority attached to the written information reduced the double-checking and correction processes we observed in the co-located setting. The continuous face-to-face presence in the same room provides the opportunity to be “*in sync*” with others’ perceived uncertainty and, thereby, interpret and correct information accordingly. The technological enhancements of the distributed setting did not entirely amend the lack of continuous face-to-face presence.

Summarized, several characteristics of the distributed setting could explain the higher degree of misunderstandings and lower SA on level 3 than in the co-located setting: the perception of text as authoritative and therefore not questioned, the lack of immediateness and continuous contact, the lack of use of the phone to check own understanding, the lack of feedback to check others’ understanding, and lastly (related to lack of immediate contact) the lack of knowing whether and what others were studying in the common operational picture.

These findings point to potential differences in how MTS integration teams are set up and used by practitioners. While integration teams could be a vital part of mutual adjustment (de Vries et al., 2021), they may have different properties that determine their usefulness. We have highlighted some implications from the communications conditions such teams operate under, where a distributed integration team could hinder rather than help integration.

In a MTS-TST setting, the task is both dynamic and complex, and what seems a routine operation at one step may develop into a largely uncertain situation in the next (Hansson et al., 2021). Moreover, the task is complex in its dynamic nature and the number of paths to the desired ends (Hærem et al., 2015). Even in more straightforward settings, such as in the aviation industry, misconceptions about situation elements arise several times during a flight (Weick, 1990; Helmreich, 2000). But the normal is that they are corrected through active interaction and sharing of information that identifies signals of danger at an early stage. The distributed setting allows for less face-to-face interaction and intense information sharing. Using richer media may prove critical to achieving a successful outcome. The ability to switch between communications media as uncertainty dynamically evolves seems to be an under-researched phenomenon in media synchronicity and MTS literature. The multiteam system setting is, by its complexity, more dynamic and varied and poses more significant challenges on the sensitivity to the shifting communications requirements of the dynamic task.

### Theoretical Implications

Prior work on MTS indicates that co-location is fruitful for creating shared understanding (e.g., de Vries et al., 2021). Yet, there is a lack of in-depth knowledge of the differences between integration among teams in distributed and co-location settings and the processes through which communications setting influence shared understanding and performance. As suggested by prior research, information sharing may not, by itself, have direct effects on performance but work through teamwork processes (Burke et al., 2006). While our findings are exploratory, they underscore the need for research to investigate processes, over time, to understand how MTS integrates information to solve dynamic and complex tasks. Our findings shed some light on the relations commented as critical to investigate in future team research by, for example, Marlow et al. (2017). This study also uncovered implications for the research on communications and adaptive team processes (e.g., Burke et al., 2006; Maynard et al., 2015). The communications condition seems to be an overlooked factor influencing key behavioral processes but can be seen as a critical contingency on MTS integration teams’ adaptive processes. One key explanatory mechanism is the mutual and immediate focus on the same objects, such as the common operational picture. No matter the sophisticated ways of keeping track of distributed participants, one may not come close to the feedback available in collocated settings.

Media richness positively influenced SA on level 3, which is in line with other experimental empirical research on media conditions and SA (Valaker et al., 2018). As predicted by Burke et al. (2006), SA led to improved performance. Still, our analysis indicates that this is only for SA on level 3 and under co-located conditions. Thus, the richness of the communications condition can be critical for the types of military tasks we studied when environmental uncertainty is high, and there is significant time pressure. Prior findings have indicated that media can pivotally influence team performance in military operations (e.g., Snook, 2002; Wilson et al., 2007; Dalenberg et al., 2009). Our study shed light on how the communications condition influences performance indirectly through the processes of establishing SA–particularly on SA level 3.

### Limitations and Future Research

This was an exploratory study, so further studies are needed to validate and generalize the findings. To gain more insight into the challenges of coordinating a MTS in a dynamic environment, studying the underlying processes of creating and sharing SA seems like a fruitful path to follow. There is a need for conceptual understanding of these processes, with corresponding operationalizations by coding of transcripts and even quantitative operationalizations. We demonstrated how one could analyze the distribution of sharedness of SA across roles and levels of awareness. However, we only scratched the surface of research questions regarding specialization and the diversity of SA in MTS. Future research could examine several questions related to this, for example: What is the SA that all team members in TST-cells need, compared to the information that can remain differentiated? How does the communications condition influence shared SA at the different levels? Extending our research illustrated in Figure 2, where we saw high co-variation between the individual roles’ SA and the team’s performance, future research could study more systematically the relation between the effect of SA differentiation and integration and performance.

Other important topics that could shed light on what influences coordination in the kind of military decision-making teams we studied: To what extent does urgency and complexity of the scenario matter? And: Do characteristics of the individuals involved, such as experience, interact with the communications condition to influence the process aspects we studied, i.e., does higher experience make distributed teams perform better?

### Practical Implications

We have discussed how the requirement for the communications condition richness changes dynamically through a TST operation based on different degrees of task complexity. Based on the theory and experiences presented in this study, choosing a communications concept that allows for the appropriate degree of communications condition richness required by the maximum possible task complexity in the scenario seems advantageous.

In a TST setting, the operation’s accuracy and speed seem to place higher demands on the communications condition than the current setup can provide. Since both the internally and externally generated uncertainty is highly dynamic in the TST setting, it is easy to end up in a situation with “underkill,” or in some cases, a missed opportunity. Furthermore, since the consequence of “underkill” often results from critical errors and persisting misunderstandings that seriously hamper both speed and accuracy of the TST process, “underkill” situations are costly, windows of opportunity may be missed, and wrong targets may be addressed.

Several strategies may be used to avoid such situations. One is to organize and train TST-cell members to be sensitive to possible errors and encourage a shift in media use when uncertainty rises. Another strategy is to prepare the cell to use face-to-face communications so that matters of low uncertainty are handled swiftly and that more resources can be allocated to address the uncertainty. It seems critical for the members of the TST-cell to use a rich communications condition to reduce uncertainty and hesitance. In other words, better late than never is not a solution.

## Data Availability Statement

The raw data supporting the conclusions of this article will be made available by the authors, without undue reservation.

## Ethics Statement

Ethical review and approval was not required for the study on human participants in accordance with the local legislation and institutional requirements. Written informed consent for participation was not required for this study in accordance with the national legislation and the institutional requirements.

## Author Contributions

All authors listed have made a substantial, direct, and intellectual contribution to the work, and approved it for publication.

## Conflict of Interest

The authors declare that the research was conducted in the absence of any commercial or financial relationships that could be construed as a potential conflict of interest.

## Publisher’s Note

All claims expressed in this article are solely those of the authors and do not necessarily represent those of their affiliated organizations, or those of the publisher, the editors and the reviewers. Any product that may be evaluated in this article, or claim that may be made by its manufacturer, is not guaranteed or endorsed by the publisher.
